# The development of guidelines for management of thyroid diseases in pregnancy – current status

**DOI:** 10.1186/1756-6614-8-S1-A11

**Published:** 2015-06-22

**Authors:** Alicja Hubalewska-Dydejczyk, Malgorzata Trofimiuk-Müldner

**Affiliations:** 1 Department of Endocrinology, Jagiellonian University, Medical College, Krakow, Poland

## 

The increasing awareness of the importance of the proper maternal thyroid function for fetal development prompted the development of guidelines on thyroid diseases in pregnancy. They have been created based on trial results as well as the personal experience of experts.

The main differences between guidelines published by the Endocrine Society (ES, 2007, revised in 2012), the American Thyroid Association (ATA, 2011) and the Polish Society of Endocrinology (PSE, 2011) are further discussed. It has to be mentioned that other national guidelines also exist.

Physiological changes in thyroid function during gestation influence test results, making the application of general population thyroid hormones reference values for pregnant women the most controversial. Commercially available free thyroid hormones assays tend to give values lower than the actual ones, particularly during the 3rd trimester of pregnancy. The Endocrine Society stresses a need to establish the trimester-specific reference values for each laboratory, however, the American Thyroid Association defines TSH reference values of 0.1-2.5, 0.2-3.0, and 0.3-0.3 µIU/ml for the 1st, 2nd and 3rd trimester of pregnancy, respectively, if such site-specific TSH reference is not available. The results of a multi-centre study on thyroid hormone reference values for Polish pregnant women are in press.

In pregnancy, there is an approximately 50% increase in daily iodine requirement. All societies recommend daily iodine intake of 250 μg during pregnancy and lactation, which should be obtained by additional supplementation with formulas containing 150 μg of iodine.

The endocrine societies are concerned with careful management of both overt and subclinical hypothyroidism in pregnant women and in women of childbearing age, especially because the number of such cases has increased significantly during the last decade. Moderate-to severe maternal hypothyreosis is associated with maternal and fetal adverse outcomes. The endocrine societies agree that a dose of L-thyroxin has to be adjusted to maintain TSH level within the trimester-specific reference range. A pregnant woman should be followed with TSH measurements every 4-6 weeks. ATA strongly recommends against therapy other than L-thyroxin preparations, including T3. After delivery L-thyroxin should be reduced to the pre-conception dose (ATA, ES, PSE) and TSH checked 4-6 weeks postponed. Consequences of mild maternal hypothyroidism, as well as the level of TSH requiring intervention, are still a matter of debate. According to current guidelines, TSH levels in hypothyroid women of childbearing age should be maintained below 2,5 µIU/ml to reduce the risk of TSH increase in early pregnancy. There is no need to monitor of the thyroid function in foetuses and newborns of hypothyroid mothers (ATA) – in Polish newborns a congenital hypothyroidism screening with TSH is routinely performed. Some issues are still debated, i.e. the necessity to treat isolated hypothyroxinemia during pregnancy or L-thyroxin administration in women with normal thyroid function and positive anti-thyroid antibodies. The lack of unequivocal data supporting the treatment of such cases during pregnancy is also stressed by the PSE.

The differences in management of hyperthyroidism during pregnancy mainly concern the use of anti-thyroid drugs: according to the ATA propylothiouracyl (PTU) should be used during the first trimester and patients treated with methimazole (MMI) should be switched to PTU at the moment the pregnancy is confirmed; following the first trimester consideration should be given to switching to MMI. The Endocrine Society recommends the use of PTU as first line therapy in the first trimester as MMI is potentially responsible for congenital abnormalities; if PTU is not available or it is not tolerated MMI should be administered. Moreover, the ES states that decision which anti-thyroid drug is given should be also driven by the practitioners’ own experience. Monitoring of the liver function in patients treated with PTU is recommended by the ES only.

The universal screening of asymptomatic pregnant women or women in childbearing age for thyroid dysfunction remains controversial. The ES guideline does not recommend the universal screening suggesting that aggressive case finding should be rather considered. However there was no agreement between all members of the Guideline Committee - some of them recommended screening of all pregnant women for serum TSH abnormalities by the ninth week of gestation or at the time of their first visit. The ATA does not recommend the universal screening. Routine TSH screening is recommended at the 4th-8th week of gestation (first prenatal visit) and in women planning pregnancy by the PSE.

Screening for thyroid autoimmunity is not recommended but may be considered if appropriate, e.g. in recurrent spontaneous abortion or miscarriage.

The frequency of nodular goitre in pregnant women is up to 20-30%, and differs between populations. The formation of new thyroid lesions during pregnancy is observed in 10-20% of women. The management of the nodular goitre in pregnancy does not differ from the general population (thyroid function assessment, a TPO in hypothyroid women, US and biopsy if needed). There are no essential differences in medullary and anaplastic thyroid cancer management in pregnant women. In case of differentiated thyroid cancer (DTC) the surgery can be performed after delivery as it is recommended by the ATA. If DTC is diagnosed before the 24th week of gestation – thyroid surgery should be considered before the end of the 2nd trimester, particularly for rapidly enlarging lesions (ES, PSE recommendation). If the patient is to be managed conservatively, L-thyroxin should be introduced to maintain the TSH level below 0.1 μIU/ml according to the PSE. The ATA recommends maintaining TSH values between 0.1-1.5 µIU/ml, and the ES – suppressed TSH and FT4 or total T4 in the upper normal range for pregnancy.

Mothers with anti-TPO positivity are at increased risk of thyroid illness after pregnancy. The discussed guidelines differ slightly in screening indications for postpartum thyroiditis. The lack of evidence for anti-thyroid drug use in thyrotoxicosis phase is unquestionable. During hypothyreosis phase careful control is necessary and the treatment with l-thyroxin of all women planning next pregnancy is recommended.

Summing up, it should be stressed that despite a huge progress which has been made in our understanding of thyroid physiology and pathology in pregnancy there are still important areas of uncertainty and further research is needed to optimize the management of thyroid diseases in pregnancy. The new version of the joint ATA and the ES guidelines is to be released in 2016. The Polish recommendations also need to be revised.
